# 

**Published:** 1880-09

**Authors:** 


					﻿Whole Number,
ACCCXXIX
September, 1880.
Number 3,
Vol. XLI.
THE
CHICAGO
MED ICAL
T ournal and Examiner
EDITORS:
N. S. DAVIS, M.D., LL.D. JAS. NEVINS HYDE, A.M., M.D.
DANIEL R. BROWER, M.D.
CHICAGO:
PUBLISHED BY THE CHICAGO MEDICAL PRESS ASSOCIATION,
188 Clark Street.
^“Read the Notice of this Journal among Advertisements.
				

